# Dietary Levels of Pure Flavonoids Improve Spatial Memory Performance and Increase Hippocampal Brain-Derived Neurotrophic Factor

**DOI:** 10.1371/journal.pone.0063535

**Published:** 2013-05-28

**Authors:** Catarina Rendeiro, David Vauzour, Marcus Rattray, Pierre Waffo-Téguo, Jean Michel Mérillon, Laurie T. Butler, Claire M. Williams, Jeremy P. E. Spencer

**Affiliations:** 1 Molecular Nutrition Group, School of Chemistry, Food and Pharmacy, University of Reading, Reading, United Kingdom; 2 School of Psychology and Clinical Language Sciences, University of Reading, Reading, United Kingdom; 3 Reading School of Pharmacy, University of Reading, Reading, United Kingdom; 4 University de Bordeaux, ISVV, Groupe d'Etude des Substances Végétales à Activité Biologique, Villenave d'Ornon, France; University of Valencia, Spain

## Abstract

Evidence suggests that flavonoid-rich foods are capable of inducing improvements in memory and cognition in animals and humans. However, there is a lack of clarity concerning whether flavonoids are the causal agents in inducing such behavioral responses. Here we show that supplementation with pure anthocyanins or pure flavanols for 6 weeks, at levels similar to that found in blueberry (2% w/w), results in an enhancement of spatial memory in 18 month old rats. Pure flavanols and pure anthocyanins were observed to induce significant improvements in spatial working memory (p = 0.002 and p = 0.006 respectively), to a similar extent to that following blueberry supplementation (p = 0.002). These behavioral changes were paralleled by increases in hippocampal brain-derived neurotrophic factor (R = 0.46, p<0.01), suggesting a common mechanism for the enhancement of memory. However, unlike protein levels of BDNF, the regional enhancement of BDNF mRNA expression in the hippocampus appeared to be predominantly enhanced by anthocyanins. Our data support the claim that flavonoids are likely causal agents in mediating the cognitive effects of flavonoid-rich foods.

## Introduction

Phytochemical–rich foods, particularly those rich in flavonoids, have been shown to be effective in reversing age-related deficits in memory and learning [1]–[6]. In particular, studies using *Camellia sinensis* (tea) [7]–[12], *Gingko Biloba* [13]–[15], *Theobroma cacao* (cocoa) [16]–[18] and *Vaccinium spp* (blueberry) [19]–[23] have demonstrated beneficial effects on memory and learning in both humans and animal models. Whilst these studies clearly demonstrate the efficacy of flavonoid-rich foods in promoting cognitive performance, they fall short of providing evidence that flavonoids themselves are the causal agents in driving beneficial effects on memory, learning and neuro-cognitive performance. Because each of these foods contains large array of macro- and micro-nutrients and a diverse phytochemical profile (flavonoids, hydroxycinnamates, phenolic acids), to date it has been difficult to assign specific biological functions to a single flavonoids or even specific flavonoid groups.

Several studies have indicated that absorbed flavonoids and their metabolites are able to transverse the blood-brain-barrier [24]–[26] and may exert neuropharmacological actions at the molecular level, influencing signalling pathways, gene expression and protein function [27]–[30]. For instance, the beneficial effects of green tea, blueberry and *Gingko Biloba* on spatial memory have been shown to involve increases in hippocampal brain-derived neurotrophic factor (BDNF) [23], [31]–[33]. The conversion of short-term memory (STM) into long-term memory (LTM) is regulated at the molecular level in neurons [34]–[36] and involves the synthesis of new proteins that control neuronal morphology and connectivity [37]. A growing body of evidence indicates that BDNF plays a key role in the regulation of both short-term synaptic function and long-term activity-dependent synaptic plasticity during memory formation [38]–[40]. Furthermore, declines in hippocampal BDNF levels occur during aging [41]–[43] and appear to negatively impact on memory performance [44], whilst both exercise and diet have been shown to influence BDNF expression in the hippocampus [45], [46].

We have previously shown that blueberry intervention induces both spatial memory improvements and BDNF signalling in young and old animals [23], [47]. However, despite evidence for the functional and molecular actions of blueberry and other flavonoid-rich foods, limited data exist with regards to the actions of pure flavonoids on memory [48]–[51]. For example, Maher et al (2006) [51] reported that administration of pure fisetin improves recognition memory in rodents, although the underpinning mechanisms were investigated *ex-vivo* in hippocampal slices. In addition, the administration of pure (−)-epicatechin has been shown to improve the retention of spatial memory in the Morris Water Maze (MWM) [50], albeit at doses above those considered to be dietary. In the present study, we have extended these studies by examining the impact of dietary quantities of pure anthocyanins and the flavanols (similar to those present in blueberry) on spatial working memory and BDNF modulation in the hippocampus of aged rats (18-month old).

## Materials and Methods

### Materials

Antibodies used were anti-GAPDH (New England Biolabs, Hitchin, UK); anti-BDNF (Santa Cruz Biotechnology, Santa Cruz, CA); anti-pro-BDNF, (Millipore, Warford, UK). Horseradish peroxidase-conjugated goat anti-rabbit secondary antibody (Sigma, UK), ECL reagent and Hyperfilm-ECL were purchased from Amersham Biosciences (Amersham, UK). Pure standards of (−)-epicatechin and (+) catechin were purchased from Sigma (Poole, UK) and standards of anthocyanins and anthocyanidins were obtained from Extrasynthese (Genay, France). HPLC-grade hexane, acetone, glacial acetic, acetonitrile, methanol, water, and hydrochloric acid were purchased from Fischer Scientific (Loughborough, UK). Standard AIN-76A purified diet for rodents was purchased from Research Diets (New Jersey, USA). All other reagents were obtained from Sigma or Merck (Poole, UK).

### Intervention diets

Diets were prepared by Research Diets Inc. (USA) by incorporating either a) blueberry powder, b) pure anthocyanins or c) pure flavanols into the standard AIN-76A purified diet for rodents (Research Diets, USA) and made into dry pellets for animal consumption. The blueberry powder was prepared as follows: whole fresh Highbush blueberries (A.G. Axon and Sons, UK) were blended, freeze dried, powdered using a miller (APEX Construction, UK), sieve size 0.027 inches, and then incorporated into the standard rodent feed (AIN-76A) at the level of 2% (w/w), similar to that previously used [22], [23]. The pure anthocyanin extract was prepared by extracting anthocyanins directly from the blueberries giving rise to a pre-purified extract, containing 62.4% anthocyanins expressed as malvidin-3-*O*-*β*-glucopyranoside equivalents (as analyzed by HPLC). Both anthocyanin and flavanol diets were prepared by incorporating either the anthocyanin extract or pure flavanols, (+)-catechin and (−)-epicatechin (Sigma, UK), into the standard rodent feed at a level equivalent to that found in the blueberry diet (2% w/w). The blueberry powder and the anthocyanin extract were analysed for their flavonoid content prior to incorporation into the diets as described previously [52]–[54].

The blueberry-supplemented feed contained approximately 253.1 µg flavonoids/g feed (179.0 µg anthocyanins; 74.1 µg flavanols). The pure anthocyanin diet contained approximately 179.0 µg of anthocyanins (69.9 µg of delphinidin-3-*O*-*β*-glucopyranoside; 12.2 µg of cyanidin-3-*O*-*β*-glucopyranoside; 34.7 µg of petunidin-3-*O*-*β*-glucopyranoside; 2.60 µg of peonidin 3-*O*-*β*-glucopyranoside; 62.6 µg of malvidin-3-*O*-*β*-glucopyranoside). The flavanol diet contained 14.8 µg of pure (−)-epicatechin and 59.3 µg of (+)-catechin. The control diet was prepared by matching the AIN-76A purified diet for the levels sugars (glucose, fructose and sucrose) and vitamin C present in the blueberry diet. No flavonoids were detected in the control diet. All diets were prepared by Research Diets Inc., USA and were iso-caloric and matched as far as possible for macro- and micro-nutrients (such as sugars and vitamins).

### Animals and supplementation

All procedures were conducted according to the specifications of the United Kingdom Animals (Scientific Procedures) Act, 1986. The programme of work, of which the experiments described here were a part, was reviewed by the University of Reading Local Ethical Review Panel and was given a favorable ethical opinion for conduct. Utmost effort was utilized to prevent suffering and minimize the numbers of rats required for this experiment. Four groups of adult, male Wistar (n = 8 per group, Harlan, UK) were housed in groups of 2 and maintained on a 12 h light-dark cycle (lights on at 10 a.m.). All rats were 18 months old at the start of the experiment. After the habituation and shaping sessions period (described below) animals were administered one of 4 diets for 6 weeks: A) Control diet; B) 2% (w/w) Blueberry diet; C) Anthocyanin Extract and D) Flavanols (−)-epicatechin and (+)-catechin. All diets were kept in a dry and dark place and administered fresh each day to the animals. Food intake was monitored daily (around 10 am) by weighting the amount of food administered to each cage and the amount remaining in the cage in the following day. Animal weight was monitored daily. During the supplementation period, all animals were tested once a week on a standard X-maze alternation task (described below).

### Spatial Memory Testing

#### Habituation and Shaping Sessions

Rats were tested in a cross-maze apparatus as described previously [55]. Extramaze cues (laboratory furniture, lights and several prominent visual features on the walls) were held constant throughout the experiment. Rats were first habituated to the maze apparatus for 4 consecutive days. During the habituation period the rats were starved overnight in order to motivate them to collect pellets of food from the maze, after which starvation overnight was ceased. Following habituation, rats received 6 weeks of shaping sessions to assure that the animals could reliably collect rewards from the end of the maze arms before testing and supplementation begun. As such, each rat received two shaping sessions per week. Each shaping session consisted of six trials. During each shaping trial, rats were trained to enter an open goal arm and collect a reward pellet from the food well of that goal arm (entry to the alternate goal arm was restricted). This process was repeated until the rat had completed 6 trials. Across each shaping session, the ‘open’ goal arm was varied between trials according to a pseudorandom schedule.

#### Alternation task

Immediately after completion of the 6-week shapping period, test sessions were started. Each test session (8 trials) contained a pseudo-random sequence of correct choices between the two arms, as well as a pseudo-random sequence for the start arm during the choice phase. All rats were un-fasted during the procedure as the reward pellets provided sufficient motivation to ensure a high level of responding in the animals. Testing sessions were performed as described previously [23] with each animal receiving 8 trials per test session, with 5 minutes interval between trials. Here, each trial consisted of a sample phase and a choice phase. During the sample phase, a rat was placed in the start arm and allowed access to only one goal arm, entry to the goal arm was rewarded with a pellet in the food well. Access to the alternate goal arm was restricted during this phase. Once the reward pellet had been collected and eaten, the sample phase was over and the animal was placed back on the start arm for 10 seconds. During the choice phase, both goal arms could be accessed and the animal was allowed to make a free choice between these 2 arms. However, the rat was only rewarded for entering the arm that it had not visited during the sample phase. An animal was deemed to have selected an arm when it had placed a hind foot down that arm; retracing before the hind foot crossed the line was measured as a failure on the task. If the animal chose the correct arm, it was rewarded with a pellet in the food well. The animal was then returned to its cage for 5 minutes, before the next trial. The maze was cleaned with 50% ethanol solution between trials to remove any olfactory clues. For each trial, choice accuracy was measured. The number of correct choices (max 8 out of 8 trials) was recorded for each testing session. Rats were given 6 test sessions in total on the cross-maze, one test session per week with the first session being administered immediately before supplementation was started (baseline). The weight of all animals was measured over course of the experiment, as well as the daily amount of food consumed. During the experiment a total of three animals died of natural causes, one from the control-diet group (leaving the control group with a total of 7 subjects) and two from the blueberry-supplemented group (leaving the blueberry group with a total of 6 subjects).

### Tissue collection

Following the final test session animals were sacrificed by decapitation and their brains were immediately extracted and halved. Half the brain was frozen in dry ice at −80°C until it was sectioned for *in situ* hybridization (see below), the remaining half brain had the hippocampus dissected out and this was frozen at −80°C until used for Western immunoblotting analysis.

### Western Immunoblotting

Proteins were extracted using the Trizol method [56], as described previously [47] and optimized for the extraction of BDNF. Protein concentration was determined by a Lowry based protein assay [57] (Bio-Rad RC DC Protein Assay) (Bio-Rad, UK). For analysis of proteins by Western Immunoblotting, samples were incubated for 2 min at 95°C in boiling buffer (final concentration: 62.5 mM Tris, pH 6.8; 2% SDS; 5% 2-mercaptoethanol; 10% glycerol and 0.0025% bromophenol blue). Samples were stored at −80°C until analysis. Six animals per group were used for the Western Blotting Analysis, representing the total number of blueberry-fed animals and six animals (out of 7/8) randomly chosen from control, anthocyanins and flavanols diet groups. Protein samples from these animals (40–80 µg/lane) were run on 9–12% SDS-polyacrylamide gels and then transferred to nitrocellulose membranes (Hybond-ECL®; Amersham) by semi-dry electroblotting (1.5mA/cm^2^), as described previously [23], [47]. Blots were then incubated with either anti-BDNF pAb (1∶1000), anti-pro-BDNF pAb (1∶1000) or anti-GAPDH pAb (1∶5000).Bands were analyzed using the band analysis software UVISoft Band. Molecular weights of the bands were calculated from comparison with pre-stained molecular weight markers (MW 27,000–180,000 and MW 6,500–45,000, BioRad) that were run in parallel with the samples. GAPDH levels were used to normalize pro-BDNF and BDNF protein levels, as such relative band intensities were calculated as a ratio of BDNF or pro-BDNF and GAPDH levels.

### Preparation of brain sections

Coronal sections (10 µm) containing the dorsal hippocampus were cut using a cryostat, Bright Cryostat model OTF (Huntingdon, UK), and mounted onto poly-L-lysine coated microscope slides (VWR, UK). Sections were fixed for 15 min in 4% paraformaldehyde in DEPC-PBS (phosphate buffered saline, pH 7.4, which had been treated overnight with 0.1% diethylpyrocarbonate and autoclaved before use), followed by two 10 min incubations in DEPC–PBS (pH 7.4). Sections were then acetylated (0.1 M triethanolamine, 0.25%acetic anhydride in DEPC-treated 0.9% NaCl), dehydrated through a graded series of ethanols (70%, 95%, 100%), delipidated for 5 min in chloroform and placed in 95% ethanol for 5 min. Sections were then air dried and stored frozen at −80°C.

### In situ hybridization riboprobes

The methodology used was adapted from that described previously in [58]. Plasmid containing a fragment of rat BDNF cDNA (460bp cloned between the EcoRI and Sph1 site of pGEM4Z) [59] was cut with *Eco*RI. The cDNA template was purified using a GFX column (GE Healthcare, UK), quantified in a NanoDrop Spectrophotometer (Thermoscientific, UK) and purity confirmed by ratio A_260_/A_280_ ≥1.8. The riboprobes (Antisense and Sense) were transcribed from cDNA template using T7 RNA polymerase and simultaneously labeled with fluoroscein-12-UTP (Boehringer Mannheim, UK). The probe was kept at −80°C. Just before being used the probe was diluted (1/500) in hybridization buffer (0.02% bovine serum albumin, 0.02% polyvinyl pyrrolidone, 0.02% ficoll, 10% dextran sulfate, 50% formamide, 50 mM polyadenylitic acid, 100 mg/ml herring sperm DNA, 600 mM NaCl, 60 mM sodium citrate, pH 7.4), incubated at 65°C for 15 min and placed on ice.

### In situ hybridization

In situ hybridization was conducted as described previously [47]. The relative mRNA levels in hippocampus and cortex were assessed by optical density measurements. Images were captured using a CCD camera AxioCam MR3 (Zeiss, UK) connected to Microscope Zeiss – Imager A1 Axio (Zeiss, UK). All the microscope parameters were kept constant for all the sections (Scaling 10X; Exposure 2.6 ms). The densiometric analysis was carried out using Image J. The optical density of the several hippocampal subfields (Dentate Gyrus- DG; Polymorphic cell layer- PCL; Cornu Ammonis 1- CA1; Cornu Ammonis3 – CA3) and cortex was measured from two sections per animal and 6 animals per group. The mean optical density from each region in each section was corrected by subtracting the mean optical density of the background. The data is presented as mean (± S.E.M) of the corrected optical density measurement within each group.

### Statistics

For the behavioral data, “choice accuracy”, defined as the number of correct choices in the X-maze, was subjected to two-way analysis of variance (ANOVA) for repeated measures with diet group (Control, Blueberry, Anthocyanins, Flavanols) and time (0,1, 2, 3, 4, 5, 6 weeks) as main factors. This was followed by post-hoc Tukey tests where appropriate. The BDNF *in situ* hybridization data was subjected to a one-way ANOVA for each brain region (DG, PCL, CA1, CA3, Cortex) with diet group (Control, Blueberry, Anthocyanins, Flavanols) as the main factor. Post-hoc Tukey tests were subsequently used to examine differences between individual treatments. For Immunoblot data, statistical comparisons were carried out using to a one-way ANOVA with diet group as the main factor. Post-hoc comparisons were made using Tukey's test. Correlation coefficients were calculated using the Pearson product-moment correlation coefficient. All the data is expressed as mean ± S.E.M and was analyzed using SPSS.

## Results

### Weight and food intake

There was no significant increase in weight among the animals over the time course of the experiment (F 12, 312 = 1.347, NS), and no difference in weight between diet groups was observed (F 1, 26 = 1.162, NS). On average, animals weighed 486.8 (±8.3) g throughout the experiment. In addition, there were no significant changes in food intake for any of the 4 diet groups (P>0.05), with the control group consuming on average 29.1 g of food per day, the blueberry group 31.2 g of food/day, the anthocyanin group 31.3 g of food/day and the flavanol group 31.2 g of food/day. On average, the blueberry-supplemented group consumed 7.89 mg/day/rat of flavonoids (5.58 mg of anthocyanins; 2.31 mg of total flavanols), the anthocyanin group consumed on average 5.60 mg/day/rat anthocyanins, and the flavanols group consumed on average 2.31 mg/day/rat flavanols (0.46 mg of (−)-epicatechin and 1.85 mg of (+)-catechin).

### Spatial Working Memory

**Figure 1:**
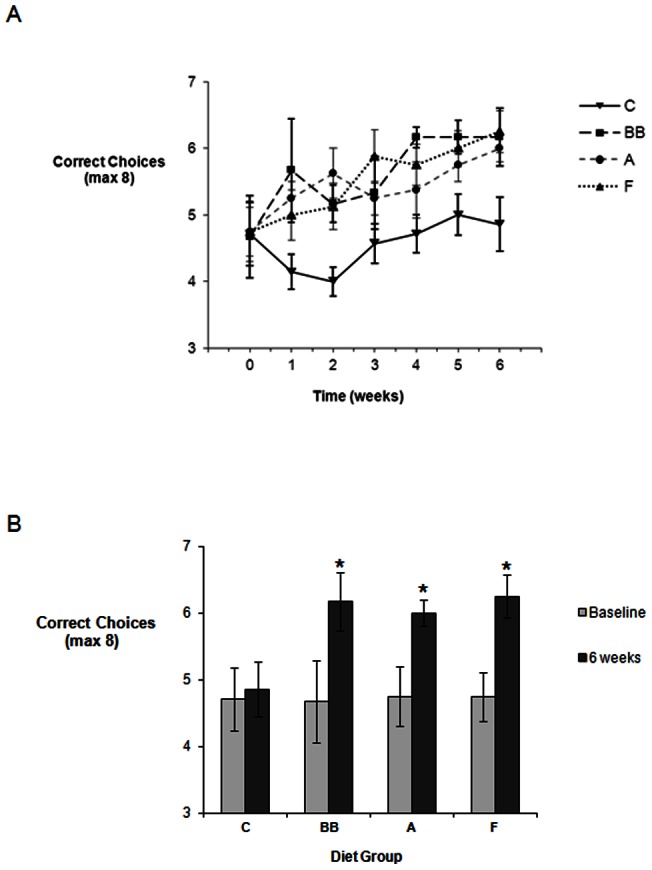
Effect of 6 weeks blueberry (BB), Anthocyanins Extract (A) and Flavanols (F) on spatial working memory in aged rats (18 months old). A) Effect of flavonoid-rich diets (BB, A or F) on correct choices (± standard error of the mean) in a X-maze Alternation Task: a significant increase in choice accuracy was observed between the control group and the blueberry group (P<0.005); the control and the anthocyanins groups (P<0.01) and the control and the flavanols groups (P<0.005). Maximum score is 8 correct choices (Control group: *Triangle ‘down’*; Blueberry group: *Square*; Anthocyanins group: *Circle*; Flavanols group: *Triangle ‘up’*). B) Comparison between animals performance at baseline and following 6 week supplementation with either a Control, Blueberry, Anthocyanins or Flavanol diet. * Indicates a significant increase in number of correct choices in comparison to baseline performance, P<0.05.

At baseline, the choice accuracy for all four diet groups was similar, showing approximately 59% accuracy (Fig. 1). There was a highly significant difference in performance between the 4 dietary groups (F 1, 25 = 7.915, P<0.001), and a highly significant effect of time (F 6, 150 = 5.354, P<0.001) but no significant interaction was seen between diet and time (F 18, 150 = 0.789, NS) (Fig. 1 A). Subsequent post hoc Tukey tests examining specific differences in performance between the individual diet groups indicated that there was a significant increase in choice accuracy between the control group and each diet (control vs. blueberry: P<0.005; control vs. anthocyanin: P<0.01; control vs. flavanol: P<0.005). Although we observed an apparent decline in performance in the control group in the first two weeks of intervention in comparison to baseline performance, this was not statistically significance (F 6, 48 = 1.239, NS). As such, the control group maintained an average score of 60% correct choices throughout the experiment, whilst the blueberry-, anthocyanin- and flavanol-diets induced an improvement in choice accuracy over the course of the intervention period, with all groups achieving between 75–80% choice accuracy by the end of the intervention period (Fig. 1B). Post-hoc analysis indicated that the significant improvements in choice accuracy observed achieved significance (in relation to baseline) by the 4^th^ week of supplementation (P<0.05), and were maintained throughout the remainder of the intervention (week 5 and 6, P<0.05) (Fig. 1B).

### Modulation of BDNF and pro-BDNF protein levels in the hippocampus

**Figure 2:**
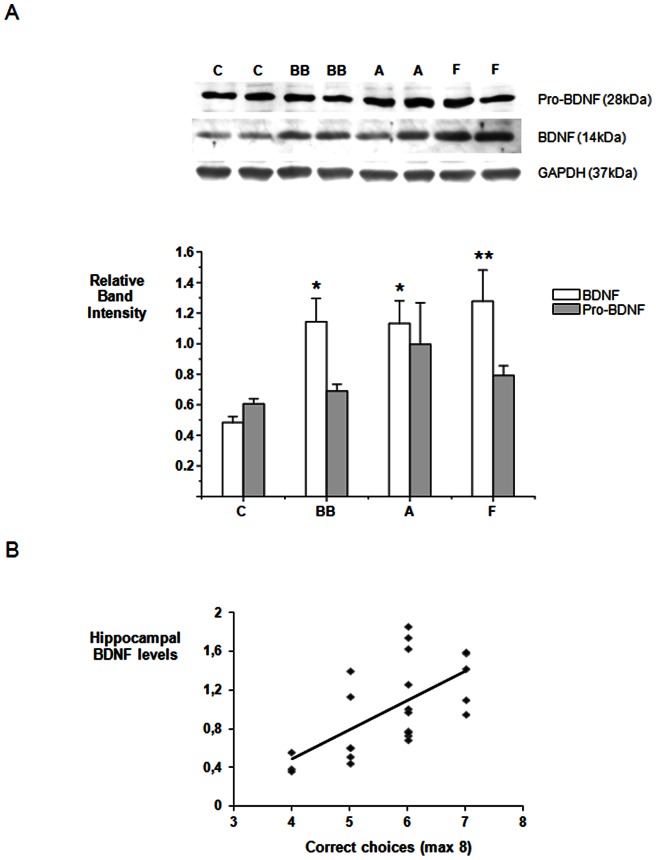
Levels of brain-derived neurotrophic factor (BDNF) in the hippocampus. A) Dissected hippocampal tissue lysates were probed for levels of pro-BDNF and BDNF using antibodies that detect the pro-domain of the BDNF protein and the mature protein. Representative immunoblots showing protein levels in 2 animals on the control diet (C), 2 animals supplemented with 2% BB diet (BB), 2 animals supplemented with Anthocyanins Extract (A) and 2 animals supplemented with Flavanols ((−) Epicatechin and (+) Catechin) (F) are presented. Pro-BDNF (grey bars) and mature BDNF (white bars). * Indicates a significant increase in BDNF levels in Blueberry and Anthocyanins groups relative to the control group, P<0.05; n = 6. ** Indicates a significant increase in BDNF levels in Flavanols group relative to control, P<0.01, n = 6. Pro-BDNF and mature BDNF were normalized against GAPDH. B) Correlation between choice accuracy (number of correct choices) in spatial memory task after 6 weeks of dietary interventions (C, BB, A and F) and levels of hippocampal BDNF protein levels, n = 24.

Levels of hippocampal pro- and mature BDNF were assessed by Western immunoblotting and normalized against GAPDH protein levels (Fig. 2A). A one way-ANOVA revealed significant differences in BDNF levels between the diet groups (F 3, 23 = 5.751, P<0.005), although no significant changes were observed for pro-BDNF (F 3,23 = 1.40, NS). Further post-hoc comparisons (Tukey test) between diet groups revealed significant increases in BDNF levels in the hippocampus of the blueberry (P<0.05), anthocyanin (P<0.05), and flavanol (P<0.01) fed groups compared to the control group. In addition, there was a significant positive correlation between hippocampal BDNF levels in individual animals from all dietary groups and their performance on the spatial memory task (R = 0.46, P<0.01) (Fig. 2B).

### Changes in hippocampal BDNF mRNA levels

**Figure 3:**
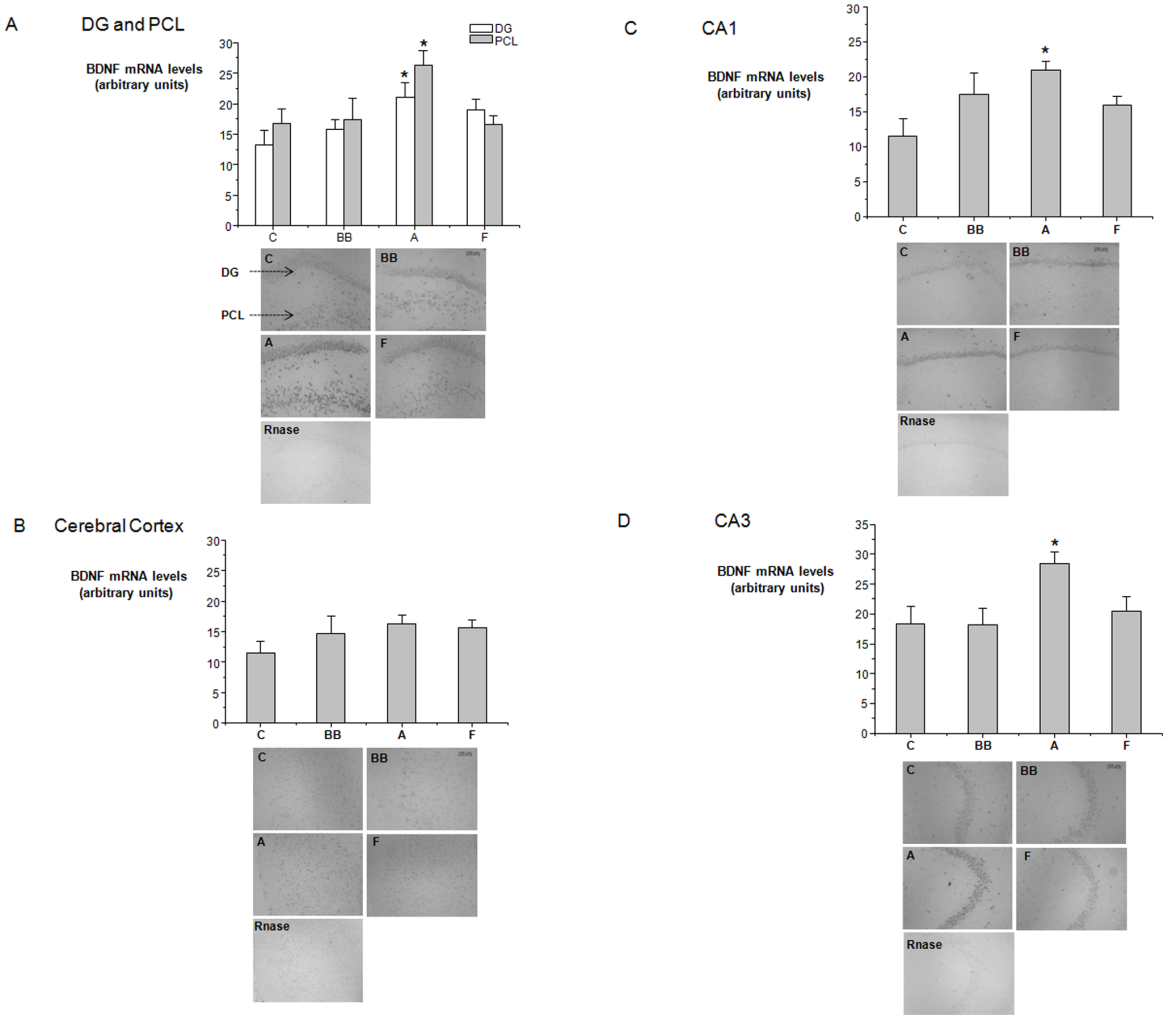
Effects of blueberry supplementation in BDNF mRNA levels in the hippocampus and cortex. **A) Dentate Gyrus (DG) (white bars) and Polymorphic Cell Layer (PCL) (grey bars) of the hippocampus, B) Cortex, C) CA1 D) CA3.** Representative pictures of hippocampal and cortical sections showing BDNF mRNA expression from 1 animal from the control (C) group, one from the BB group (BB), one from the Anthocyanins group (A) and one from the Flavanols group (F) are presented. * Indicates a significant increase in BDNF mRNA levels in the anthocyanins group in comparison to control in DG, PCL, CA1 and CA3, P<0.05. No significant differences between the four diet groups were observed in the cerebral cortex. Optical density levels are shown as mean ± SEM derived from at least 6 animals per group. Representative Rnase treated sections are presented for each hippocampal region. The scale presented represents 100 µm.

The hybridization pattern obtained for the BDNF probe was similar to that detected previously [60], with all the principal hippocampal layers exhibiting BDNF mRNA expression, including the dentate granule cell layer in the dentate gyrus. On RNase pre-treated sections, the hybridization signal was very low or absent, with RNase treated sections significantly lower in signal than the non-treated sections for all regions in the hippocampus and in cortex (P<0.001) (Fig. 3). An initial one-way ANOVA revealed that there was a significant difference in BDNF mRNA levels between the 4 diets in the dentate gyrus (F 3,24 = 3.293, P<0.05) (Fig. 3A), PCL (F 3, 24 = 3.925, P<0.05) (Fig. 3A), CA1 (F 3, 25 = 3.460, P<0.05) (Fig. 3C) and CA3 region (F 3,24 = 3.868, P<0.05) (Fig. 3D). In contrast, no significant changes in BDNF mRNA expression were detected in the cortex (F 3, 25 = 1.218, NS) following intervention with any of the 4 diets (Fig. 3B). Post-hoc analysis revealed that only the pure anthocyanin intervention led to a significant elevation of BDNF mRNA expression relative to the control group, and that this increase was observed in the DG, PCL, CA1 and CA3 hippocampal regions (P<0.05) (Fig. 3). The greatest anthocyanin-induced increase was observed in the in CA1 region, which exhibited an approximate 81% increase in BDNF mRNA expression compared to the control group (Fig. 3C).

Whilst the blueberry and the pure flavanol diet failed to significantly increase hippocampal levels of BDNF mRNA in the DG, PCL and CA3 regions, both were observed to increase BDNF expression relative to control in the CA1 region, by about 52% and 38% respectively, albeit non-significantly (Fig. 3C). Despite the fact that BDNF mRNA changes in response to blueberry and flavanols were non-significant, we observed significant correlations between hippocampal BDNF protein levels and BDNF mRNA levels in all of the hippocampal regions: DG (R = 0.59, P<0.01), CA1 (R = 0.55, P<0.01) and CA3 (R = 0.46, P<0.05).

## Discussion

Flavonoid-rich foods such as blueberry, green tea and *Gingko biloba* have been shown to be highly effective at reversing age-related deficits in spatial memory and in the enhancement of different aspects of synaptic plasticity, [19], [32], [61]–[63], a process severely affected by ageing [64], [65]. For example, we have previously shown that intervention with a 2% (w/w) blueberry diet resulted in significant improvements in spatial working memory that were mediated through the activation of the ERK-CREB-BDNF pathway, a pivotal pathway for the control of synaptic plasticity [23]. In the present study, we have shown that dietary levels of pure flavanol monomers (−)-epicatechin and (+)-catechin and a pure anthocyanin mixture (reflective of those found in blueberries) are also capable of mediating improvements in spatial working memory in aged animals. Indeed, the changes in spatial memory induced by the pure flavonoids mimicked those induced by whole blueberry, suggesting that the flavonoids are likely to be responsible for the efficacy induced by the whole fruit *in vivo*.

These findings were supported by observations that enhancements in spatial memory induced by the flavonoid-rich diets also significantly correlated with increases in hippocampal BDNF protein levels, suggesting that the effect of flavonoids on this neurotrophin may underpin performance on memory tasks. Our data agree with previous findings indicating that (−)-epicatechin dosed at 125 mg/kg of body weight (BW) per day for 6 weeks, is capable of enhancing synaptic plasticity; a crucial process during spatial learning [50]. However, our data indicates that such changes in spatial memory are also induced by significantly lower doses of flavonoids, more reflective of normal dietary intake [Human equivalent dose (mg/kg): flavanols  = 4.75 mg/kg ×(6/37)  = 0.77 mg/kg or 54 mg for a 70 Kg adult; anthocyanins  = 130 mg for a 70 Kg adult] [66].

There is solid evidence indicating that hippocampal BDNF expression, in response to spatial memory training, is associated with memory performance [44], [67], [68]. BDNF has been shown to play a crucial role in synaptic plasticity, where it controls the stability of the hippocampal circuitry through its action in promoting changes in neuronal spine density and morphology [69]–[71]. Such morphological changes, stimulated by BDNF, dictate the efficiency of the synaptic connections and consequently affect spatial learning outputs [72]. Additionally, increases in neurotrophin expression may be also be important in determining neurogenesis [50], with some data suggestive that BDNF plays an important role in modelling the neurovascular niche, particularly in the formation and maintenance of the vascular tube, which affects new neuronal proliferation and differentiation [73], [74].

The elevation of hippocampal protein levels of BDNF by blueberry and pure flavonoids is particularly relevant as BDNF expression in the hippocampus is known to decrease with age in several mammalian species, including humans [42], [43], [75], with these decreases associated with a decline in spatial memory [41], [44], [76]. Furthermore, these age-related alterations in BDNF expression appear to be region-and circuit specific [77], [78]. We observed the greatest regulation of BDNF mRNA expression by pure anthocyanins in the CA1 region of the hippocampus, although levels were significantly increased in all hippocampal regions assessed. In contrast, blueberry and flavanol interventions did not appear to affect mRNA BDNF expression, despite the increases in protein levels detected in the hippocampus. Despite this, we observed a significant correlation between hippocampal protein levels of BDNF and levels of BDNF mRNA in the DG, CA1 and CA3 regions following intervention with all three of the flavonoid-containing diets, suggesting that the changes in BDNF protein are at least partly dependent on flavonoid-induced BDNF mRNA expression.

Alternatively, flavanols present in the blueberry diet may act to increase hippocampal BDNF levels via alternative mechanisms, such as through BDNF stabilization rather than its *de novo* synthesis. BDNF protein levels in the neurons originate from the cleavage of pro-BDNF into mature BDNF by the tPA/plasmin enzyme system, which is expressed at the hippocampal synapses [79]. Spatial learning is known to strongly increase the conversion of pro-BDNF into BDNF in both young and aged animals, with this process being typically down-regulated by ageing. As such, changes in BDNF protein levels in neurons do not always directly reflect changes in BDNF mRNA levels [80]. The increased expression of hippocampal protein BDNF levels seen after intervention with pure flavanols may be mediated by increases pro-BDNF metabolism during learning rather than via increases in pro-BDNF mRNA expression. In support of this, we observed lower levels of pro-BDNF in the flavanol group in comparison to the anthocyanin group (albeit not significantly), suggesting increased pro-BDNF metabolism during learning for the flavanol group.

Previously, we have observed an increase of mRNA BDNF in different regions of the hippocampus in young healthy animals, followed by an increase in both pro-BDNF and BDNF protein levels, suggesting that the flavonoids present in blueberry have the potential to stimulate both BDNF expression as well as BDNF stabilization [47]. Since in an aged rat both these processes are typically down regulated [81], the data emanating for this study suggest that flavanols given in a pure form are more efficient in regulating BDNF metabolism/stabilization during learning whilst pure anthocyanins may play an important role at stimulating *de novo* BDNF expression. However, a direct comparison between the effects of flavonoids in these two experiments is not trivial as the rats species used were different and age-dependent changes in BDNF are known to differ among rat species [81]. Thus, such an analysis would be valuable in future work to better understand how age differences impact on the potential effects of flavonoids on brain health.

Although, the mechanisms by which flavonoids act in the brain are not clear, there is evidence to suggest that blueberry flavonoids can cross the blood-brain barrier (BBB) and reach the central nervous system, where they have the potential to directly regulate gene and protein expression in neurons [23], [24], [82]. However, at present it is unclear as to whether flavonoid-induced memory improvements are mediated exclusively centrally or whether other mechanisms such as stimulations in endothelial function and peripheral blood flow [83] also contribute. Such vascular effects are significant since it has been reported that increased cerebrovascular blood flow facilitates proliferation of neuronal cells in the hippocampus and this may influence memory [84].

Our study presents evidence that dietary quantities of pure flavanols, (−)-epicatechin and (+)-catechin and pure anthocyanins are capable of inducing beneficial effects on memory in aged rats. As such, our data add weight to the evidence suggesting that flavonoids are the causal agents in determining the cognitive benefits of flavonoid-rich foods such as blueberry. Our data further support the view that such effects of flavonoids are determined at the molecular level in the hippocampus, where they are able to increase the expression of BDNF in specific regions of the hippocampus. Most notably, our data suggest that dietary amounts of flavanols and anthocyanins are capable of inducing both molecular and behavioral changes linked to memory in rats. As such, these compounds represent potential therapeutics that can counteract age-associated cognitive decline through dietary intervention or most importantly can play a crucial role in preventing age-related cognitive impairment.
